# Secondary Hemorrhage from Gun-Shot Wounds, with a Case

**Published:** 1863-11

**Authors:** Wm. H. Gail

**Affiliations:** Medical Cadet, U. S. A.


BUFFALO

### Medical and Surgical Journal.

VOL. III. NOVEMBER, 1863. NO. 4.

### ORIGINAL COMMUNICATIONS.

ART. I.—*Secondary Hemorrhage from Gun-Shot Wounds, with a case.* By Wm. H. Gail, *Medical Cadet, U. S. A.*

Secondary hemorrhage from gun-shot wound, says Mr. Roux, proceeds from "separation of the eschar; from injury by fractured bones; from the capillaries caused by general feebleness of the patient." The latter corresponding with the "hemorrhagic diathesis" of Druit, produced by long campaigns, improper diet, and the absence of a sufficient quantity of vegetable food, to prevent the system from laboring under a scorbutic cachexa. But whatever be the cause, it is a disagreeable complication, and requires prompt and energetic action to insure any degree of safety to the patient. Stronmeyer relates a case, showing the necessity of knowing what to do, and the presence of mind, to do it without delay.

An arterial hemorrhage from a wounded brachial artery, occurred in the third week of the injury—a fracture of the humerus; the arm was amputated, but the patient soon died, having lost so much blood in the first instance; "an incompetent medical man who was present not having presence of mind to employ compression." In the treatment of this *accidental* complication of gun-shot injuries, the first indication is, to arrest the hemorrhage; the second, to prevent its recurrence. The first may be fulfilled by compression, ice, persulphate of iron; these, if the artery be small, and the hemorrhage be slight, may fulfill the second indication also; but if a large artery be implicated, deligation is necessary, and we have to choose between the rule promulgated by Bell and Guthrie, of tying both extremities of the divided arteries; that of Anel, who advocates the ligation of the main trunk at a distance from the wound, between it and the heart, and amputation. In primary hemorrhages the plan of tying the divided extremities is preferable; but in hemorrhages occurring after the limb has become swollen, its tissues infiltrated, agglutinated and disorganized, to cut down and deligate the extremities of the bleeding vessel, is a task not easily performed. Experience has proved Anel's operation to be unsatisfactory in its results, (vide McLeod's notes on Surgery of the Crimean War, page 135, and note on page 136,) and consequently amputation is resorted to.

The following case will illustrate the difficulty that would have been experienced had an attempt been made to perform Guthrie's operation; and, although it is not positively disparaging to the method advocated by Anel, its result cannot with propriety be considered to favor it.

Sergeant James Furguson, Co. G, 142d Pennsylvania Volunteers, a young man of good constitution. was admitted to Stanton Hospital December 29, 1862, on account of a gun-shot wound of the right leg, received in the battle of Fredericksburg December 13, 1862. The bullet passed through the calf of the leg in its upper third, behind the tibia and fibula, in a downward and outward direction. The wound did well until the middle of January, when the granulations assumed an unhealthy appearance, and the discharge became thin and serous. He also exhibited typhoid symptoms, having a hot skin, frequent pulse and dry, red tongue, watchfulness, and no appetite. In this way he went on from January 15th until Friday morning, January 23d, when hemorrhage unexpectedly occurred from the external orifice, behind the fibula. The officer of the day readily controlled bleeding by the application of pressure by a bandage, and ice. He thought the patient lost in all about 10 ounces of blood. Through that day and night the loss of blood by oozing was very little. On Saturday morning, January 24th, the bleeding recurred; this time from the internal orifice, behind the tibia. Dr. Mursick, his attending surgeon, was in the ward when the bleeding commenced; he readily controlled it by the application of persulphate of iron, lint, ice and bandages, He lost this time from 4 to 6 ounces of blood, not more than the last figure. Meanwhile the typhoid symptoms became more marked; he complained of great tenderness throughout the leg and thigh; the inguinal glands were somewhat swollen and tender; there was dusky redness with soreness in the track of the long saphenous vein; his skin was of a pale yellow hue, and he presented other symptoms of pyæmia.

On Sunday morning a slight bleeding occurred from the internal wound, which was controlled by pressure and ice; there was a marked increase of swelling of the leg noticed that morning, and infiltration thereof, with blood was suspected; the swelling was extending from the leg to the thigh, especially over the external and internal condyles, and the popliteal space also was already filled up with the swelling. He was verv pale, expressed a great deal of anxiety; pulse 120 per minute, quick and weak.

On Friday we thought hemorrhage came from the peroneal artery, on Saturday from the posterior tibial, at all events we were uncertain on Sunday morning with regard to the source of the bleeding. The case now presented an unpromising appearance on account of the debility from the loss of about 18 ounces of blood, in all not more than that, superadded to typhoid disease, or condition, and it was decided to tie the femoral artery at the apex of Scarpa's space, as affording the best chance for prolonging life. The operation was performed by Surgeon John A. Lidell on the afternoon of that day, Sunday, January 25, 1863, forty-three days after he had been wounded. The patient was manifestly pyæmic, and we scarcely hoped for his recovery on that account,

Monday morning, January 26th, patient appeared brighter, pulse 130, tongue more moist, leg getting warm down to the ankle, plugs removed from wounds, and some dark, offensive blood flowed away; 6 o'clock P. M. foot cold, leg cooler, blackness extending across the leg in track of wound, tongue dry; has had a slight chill, and he is somewhat delirious; pnlse 130 and weak.

Tuesday, January 27th, in the morning the patient looked better than he did the evening before; pulse 132, and stronger; leg warmer and blacker, foot pale and swollen, (serous inflammation;) discoloration and serous infiltration extending up the thigh.

Wednesday, January 28th, patient presents a pale, yellow hue; blackness of the limb deepening and extending; has reached the lower end of the incision for tying femoral artery; odor gangrenous.

Thursday, January 29th, 1863, patient sank, and died in the evening, four days subsequent to the deligation of the femoral artery.

Autopsy showed that the bleeding came, not from the posterior tibial or peroneal artery, as had been supposed, but from the lower part of the popliteal artery, which had been open to a large extent by ulceration; it was also found that the ball had grazed the hind part of both the tibia and fibula in its track, and there were some loose splinters, small in size, in relation with those bones.

A piece of the femoral artery, four inches long, was taken out, which embraced the seat of deligation. On the proximal side of the ligature the clot was firm, closely attached to the walls of the artery, and about three-fourths of an inch in length, with its apex pointing upward toward the heart. On the distal side of the ligature there was also a firm clot adherent to the walls of the artery, but this clot was much smaller than the proximal one.

In this case, as frequently occurs, the patient was averse to amputation, and as his symptoms were anything but favorable to recovery, his request was acceded to, and Anel's operation performed, with the hope of prolonging life for a brief period only.

